# The genome sequence of the orange-striped anemone, *Diadumene lineata* (Verrill, 1869)

**DOI:** 10.12688/wellcomeopenres.17763.1

**Published:** 2022-03-15

**Authors:** Christine Wood, John Bishop, Joanna Harley, Robert Mrowicki, Marine Biological Association Genome Acquisition Lab, Darwin Tree of Life Barcoding collective, Wellcome Sanger Institute Tree of Life programme, Wellcome Sanger Institute Scientific Operations: DNA Pipelines collective, Tree of Life Core Informatics collective, Darwin Tree of Life Consortium

**Affiliations:** 1 Marine Biological Association, Plymouth, Devon, UK; 2 Natural History Museum, London, UK

**Keywords:** Diadumene lineata, orange-striped anemone, genome sequence, chromosomal, Cnidaria

## Abstract

We present a genome assembly from an individual *Diadumene lineata* (the orange-striped anemone; Cnidaria; Anthozoa; Actiniaria; Diadumenidae). The genome sequence is 313 megabases in span. The majority of the assembly (96.03%) is scaffolded into 16 chromosomal pseudomolecules. The complete mitochondrial genome was also assembled and is 17.6 kilobases in length.

## Species taxonomy

Eukaryota; Metazoa; Cnidaria; Anthozoa; Hexacorallia; Actiniaria; Nynantheae; Diadumenidae; Diadumene; *Diadumene lineata* (Verrill, 1869) (NCBI:txid1789172).

## Background

The Orange-striped anemone, *Diadumene lineata* (Verrill, 1869), is believed to be the world's most widely distributed sea anemone. Native to the Northwest Pacific, it is now established on almost every temperate and tropical coast worldwide, and is a remarkable colonising species that serves as a model by which to address invasion hypotheses ( Flenniken, 2017). In the UK, it has been recorded all along the south coast of England, around the Welsh coast, and from a few sites in Northern Ireland and Scotland. In these areas, it is typically found in sheltered estuaries attached to artificial structures in marinas and harbours, often in association with oysters and mussels, but also on sheltered natural shores, on stones, shells and seaweeds.

*Diadumene lineata* is a small, delicate anemone, with a smooth column up to 20 mm in diameter (in the UK, but larger in its native range). Generally, it is olive green or brown with contrasting orange vertical stripes. It has 25–100 slender, smooth tentacles, which are all of one type and usually colourless, but can be reddish. Thread-like defensive organs (acontia) can extend through pores in the column. It preys mainly on small crustaceans but may also consume larvae of commercially important species such as oysters and mussels. Under suitable conditions, it can quickly form large clonal aggregations.

In its native range *D. lineata* reproduces both asexually by fission and sexually ( Ryan & Miller, 2019). However, outside its native range it is presumed that only asexual reproduction occurs, as no populations with both males and females together have been reported, except for a recently discovered population with both males and females in Coos Bay, Oregon, USA ( Newcomer *et al.*, 2019).

## Genome sequence report

**Figure 1.**
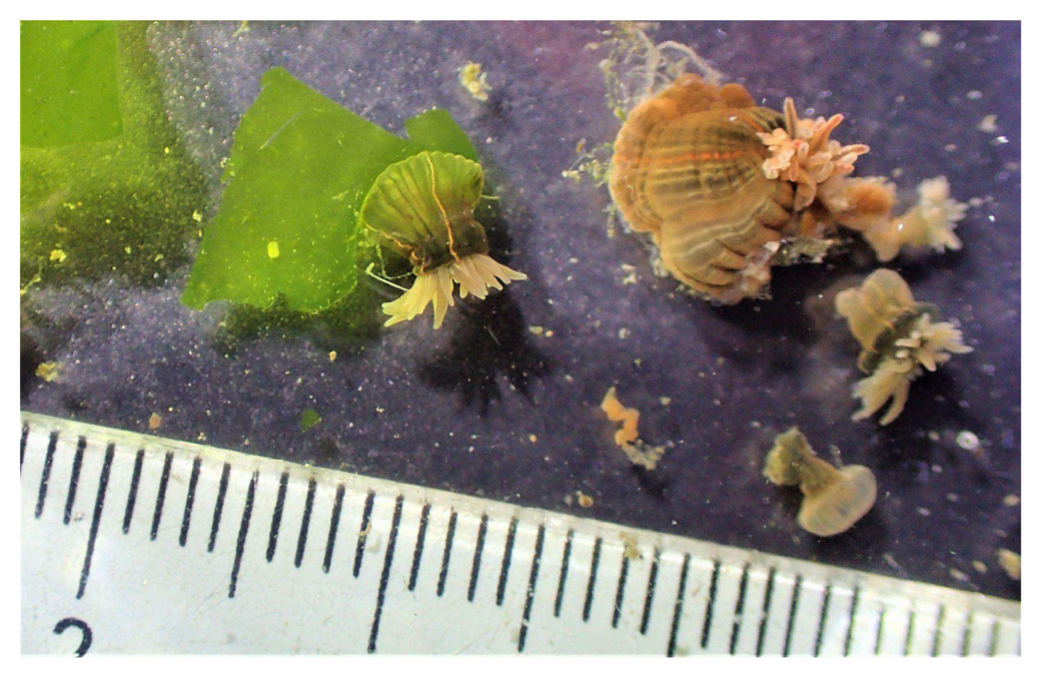
Image of the *Diadumene lineata* specimen taken prior to preservation and processing. The sample is shown in focus, slightly to the left of centre.

The genome was sequenced from a single *D. lineata* of unknown sex collected from Queen Anne's Battery Marina visitors' pontoon, Plymouth, UK ( Figure 1). A total of 27-fold coverage in Pacific Biosciences single-molecule HiFi long reads and 82-fold coverage in 10X Genomics read clouds were generated. Primary assembly contigs were scaffolded with chromosome conformation Hi-C data. Manual assembly curation corrected 113 missing/misjoins and removed 43 haplotypic duplications, reducing the assembly size by 1.34% and the scaffold number by 41.95%, and increasing the scaffold N50 by 101.80%.

**Figure 2.**
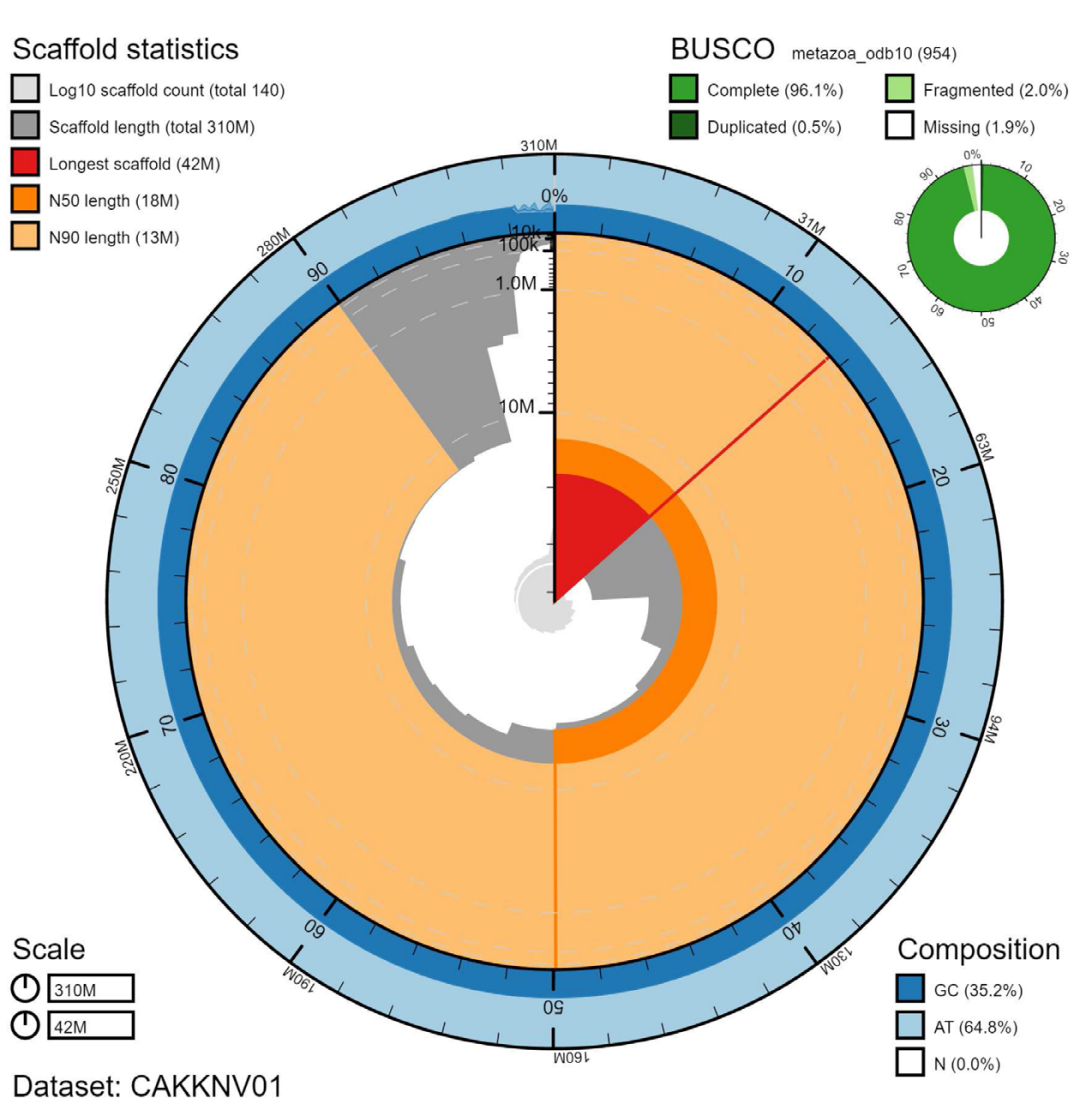
Genome assembly of *Diadumene lineata*, jaDiaLine6.1: metrics. The BlobToolKit Snailplot shows N50 metrics and BUSCO gene completeness. The main plot is divided into 1,000 size-ordered bins around the circumference with each bin representing 0.1% of the 313,006,248 bp assembly. The distribution of chromosome lengths is shown in dark grey with the plot radius scaled to the longest chromosome present in the assembly (42,014,270 bp, shown in red). Orange and pale-orange arcs show the N50 and N90 chromosome lengths (17,670,263 and 13,127,821 bp), respectively. The pale grey spiral shows the cumulative chromosome count on a log scale with white scale lines showing successive orders of magnitude. The blue and pale-blue area around the outside of the plot shows the distribution of GC, AT and N percentages in the same bins as the inner plot. A summary of complete, fragmented, duplicated and missing BUSCO genes in the metazoa_odb10 set is shown in the top right. An interactive version of this figure is available at https://blobtoolkit.genomehubs.org/view/jaDiaLine6.1/dataset/CAKKNV01/snail.

The final assembly has a total length of 313 Mb in 137 sequence scaffolds with a scaffold N50 of 17.7 Mb ( Table 1). The majority, 96.03%, of the assembly sequence was assigned to 11 chromosomal-level scaffolds, representing 16 autosomes (numbered by sequence length) ( Figure 2– Figure 5; Table 2). Two 3-Mbp sub-chromosome sized scaffolds were added as S17 and S18 to the unlocalised sequences. S17 and S18 are part of the host, as evidenced by SSU markers and coverage. Parts of the centromere could not be uniquely assigned to chromosomes and are part of the unlocalised sequence.

**Table 1.** Genome data for *Diadumene lineata*, jaDiaLine6.1.

*Project accession data*	
Assembly identifier	jaDiaLine6.1
Species	*Diadumene lineata*
Specimen	jaDiaLine6 (genome assembly); jaDiaLine7 (Hi-C, RNA-Seq)
NCBI taxonomy ID	1789172
BioProject	PRJEB46855
BioSample ID	SAMEA7536572
Isolate information	Whole organism (jaDiaLine6); other somatic tissue (jaDiaLine7)
*Raw data accessions*	
PacificBiosciences SEQUEL II	ERR6808024
10X Genomics Illumina	ERR6688656-ERR6688659
Hi-C Illumina	ERR6688655
PolyA RNA-Seq Illumina	ERR6688660
*Genome assembly*	
Assembly accession	GCA_918843875.1
*Accession of alternate haplotype*	GCA_918843945.1
Span (Mb)	313
Number of contigs	320
Contig N50 length (Mb)	2.7
Number of scaffolds	137
Scaffold N50 length (Mb)	17.7
Longest scaffold (Mb)	42.0
BUSCO * genome score	C:96.1%[S:95.6%,D:0.5%], F:2.0%,M:1.9%,n:954

*BUSCO scores based on the metazoa_odb10 BUSCO set using v5.1.2. C= complete [S= single copy, D=duplicated], F=fragmented, M=missing, n=number of orthologues in comparison. A full set of BUSCO scores is available at https://blobtoolkit.genomehubs.org/view/jaDiaLine6.1/dataset/CAKKNV01/busco.

**Table 2.** Chromosomal pseudomolecules in the genome assembly of *Diadumene lineata*, jaDiaLine6.1.

INSDC accession	Chromosome	Size (Mb)	GC%
OU974069.1	1	42.01	35.3
OU974070.1	2	33.73	35.3
OU974071.1	3	23.14	35.6
OU974072.1	4	19.48	35.2
OU974073.1	5	18.71	35.4
OU974074.1	6	18.65	35.3
OU974075.1	7	17.67	35.4
OU974076.1	8	15.82	35.3
OU974077.1	9	15.30	35.3
OU974078.1	10	14.93	35.3
OU974079.1	11	14.14	35.2
OU974080.1	12	14.10	35.2
OU974083.1	13	13.13	35.1
OU974081.1	14	13.25	35.3
OU974082.1	15	13.16	35.4
OU974084.1	16	12.63	35.0
OU974085.1	MT	0.02	37.4
-	Unplaced	13.14	33.4

**Figure 3.**
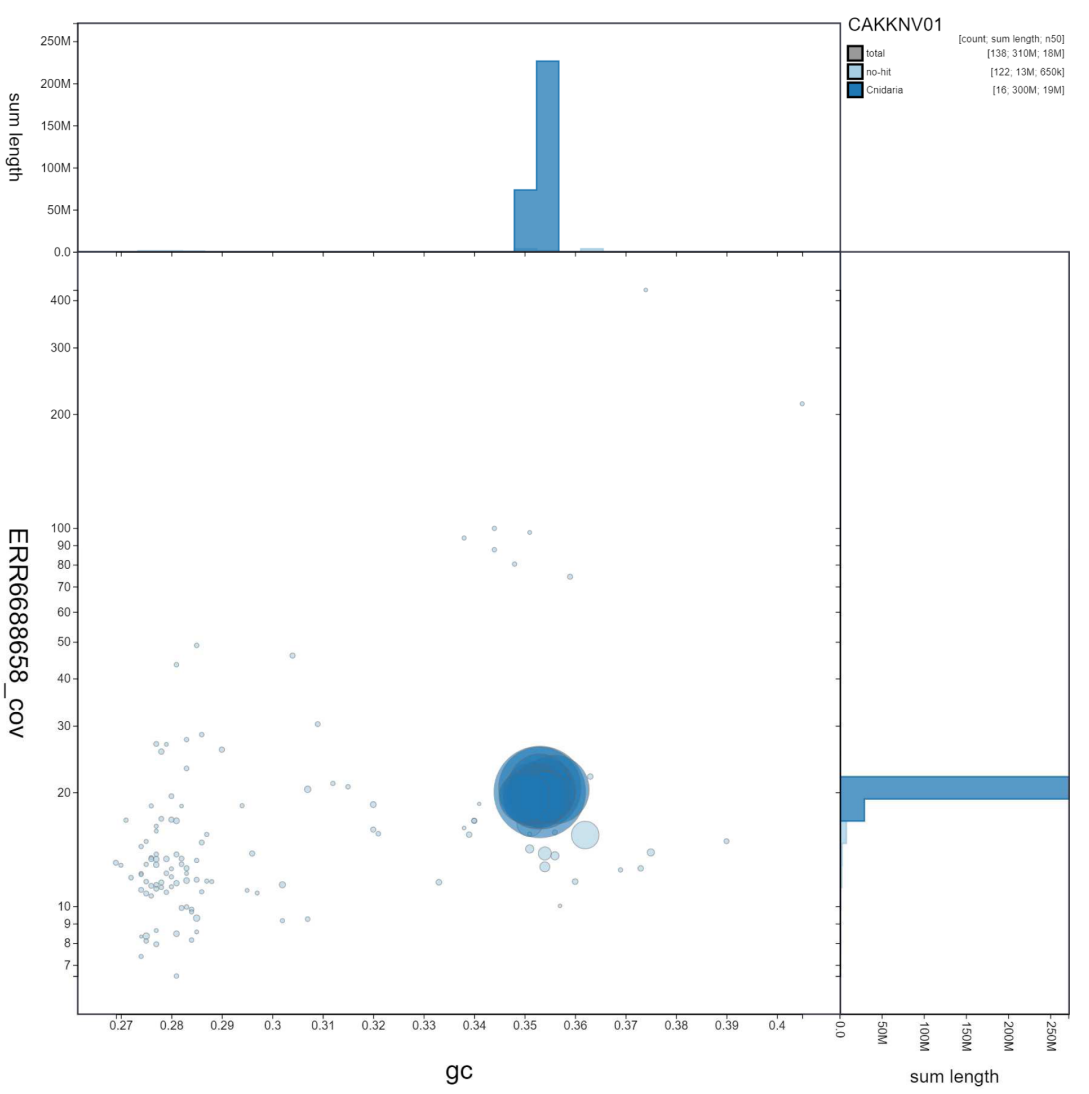
Genome assembly of *Diadumene lineata*, jaDiaLine6.1: GC coverage. BlobToolKit GC-coverage plot. Scaffolds are coloured by phylum. Circles are sized in proportion to scaffold length. Histograms show the distribution of scaffold length sum along each axis. An interactive version of this figure is available at https://blobtoolkit.genomehubs.org/view/jaDiaLine6.1/dataset/CAKKNV01/blob.

**Figure 4.**
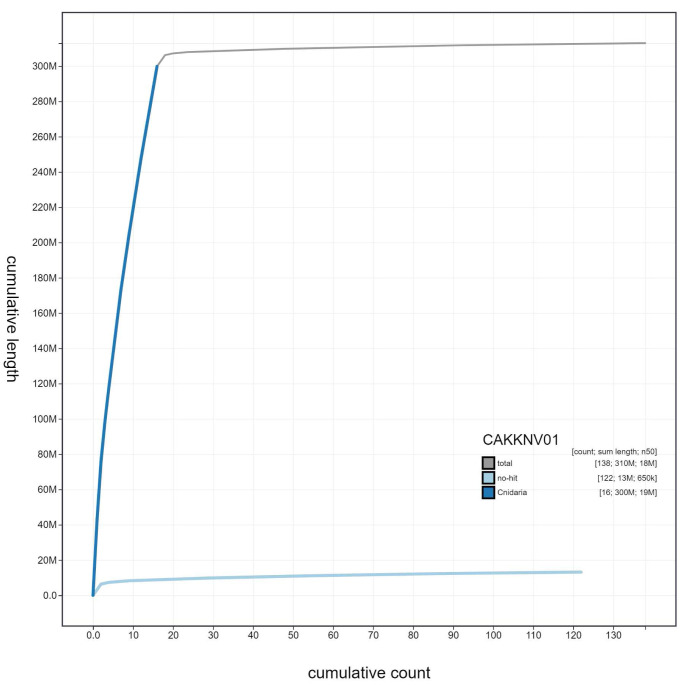
Genome assembly of *Diadumene lineata*, jaDiaLine6.1: cumulative sequence. BlobToolKit cumulative sequence plot. The grey line shows cumulative length for all scaffolds. Coloured lines show cumulative lengths of scaffolds assigned to each phylum using the buscogenes taxrule. An interactive version of this figure is available at https://blobtoolkit.genomehubs.org/view/jaDiaLine6.1/dataset/CAKKNV01/cumulative.

**Figure 5.**
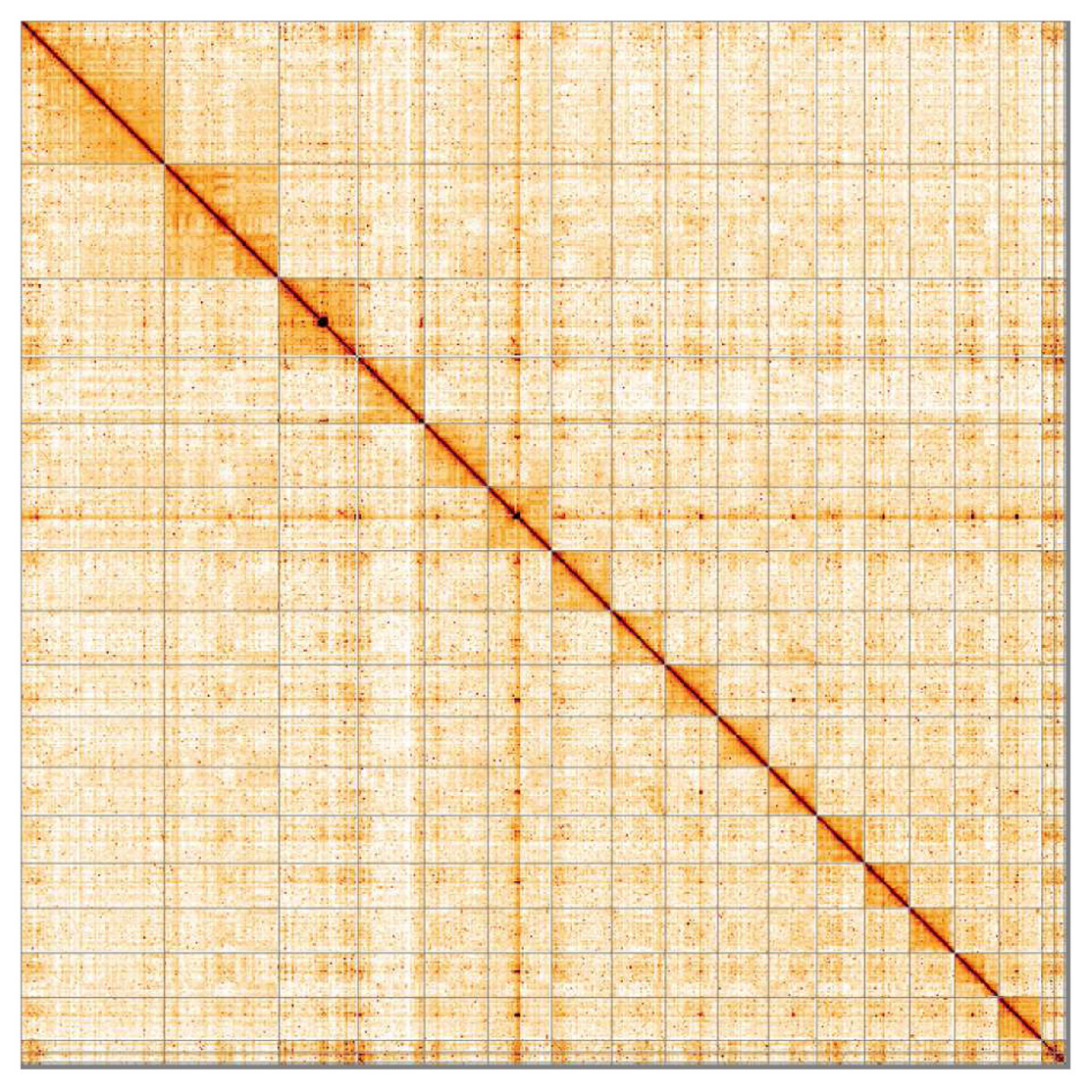
Genome assembly of *Diadumene lineata*, jaDiaLine6.1: Hi-C contact map. Hi-C contact map of the jaDiaLine6.1 assembly, visualised in HiGlass. Chromosomes are arranged in size order from left to right and top to bottom. The interactive Hi-C map can be viewed at https://genome-note-higlass.tol.sanger.ac.uk/l/?d=bZPq5k_oTFuM-wZyPAZiXg under track jaDiaLine6.

The assembly has a BUSCO v5.1.2 ( Manni *et al.*, 2021) completeness of 96.1% (single 95.6%, duplicated 0.5%) using the metazoa_odb10 reference set (n=954). While not fully phased, the assembly deposited is of one haplotype. Contigs corresponding to the second haplotype have also been deposited.

## Methods

### Sample acquisition and DNA extraction

Two *D. lineata* specimens (jaDiaLine6 and jaDiaLine7) were collected by hand from Queen Anne's Battery Marina visitors' pontoon, Plymouth, UK (latitude 50.3644, longitude -4.1320) by John Bishop, Joanna Harley (both Marine Biological Association) and Rob Mrowicki (Natural History Museum). The specimens were identified by Chris Wood (Marine Biological Association) and John Bishop and snap-frozen in liquid nitrogen.

DNA was extracted at the Tree of Life laboratory, Wellcome Sanger Institute. The jaDiaLine6 sample was weighed and dissected on dry ice with tissue set aside for Hi-C sequencing. Whole organism tissue was disrupted using a Nippi Powermasher fitted with a BioMasher pestle. Fragment size analysis of 0.01–0.5 ng of DNA was then performed using an Agilent FemtoPulse. High molecular weight (HMW) DNA was extracted using the Qiagen MagAttract HMW DNA extraction kit. Low molecular weight DNA was removed from a 200-ng aliquot of extracted DNA using 0.8X AMpure XP purification kit prior to 10X Chromium sequencing; a minimum of 50 ng DNA was submitted for 10X sequencing. HMW DNA was sheared into an average fragment size between 12–20 kb in a Megaruptor 3 system with speed setting 30. Sheared DNA was purified by solid-phase reversible immobilisation using AMPure PB beads with a 1.8X ratio of beads to sample to remove the shorter fragments and concentrate the DNA sample. The concentration of the sheared and purified DNA was assessed using a Nanodrop spectrophotometer and Qubit Fluorometer and Qubit dsDNA High Sensitivity Assay kit. Fragment size distribution was evaluated by running the sample on the FemtoPulse system.

RNA was extracted from jaDiaLine7 in the Tree of Life Laboratory at the WSI using TRIzol, according to the manufacturer’s instructions. RNA was then eluted in 50 μl RNAse-free water and its concentration RNA assessed using a Nanodrop spectrophotometer and Qubit Fluorometer using the Qubit RNA Broad-Range (BR) Assay kit. Analysis of the integrity of the RNA was done using Agilent RNA 6000 Pico Kit and Eukaryotic Total RNA assay.

### Sequencing

Pacific Biosciences HiFi circular consensus and 10X Genomics Chromium read cloud sequencing libraries were constructed according to the manufacturers’ instructions. Sequencing was performed by the Scientific Operations core at the Wellcome Sanger Institute on Pacific Biosciences SEQUEL II (HiFi), Illumina NovaSeq 6000 (10X) and Illumina HiSeq 4000 (RNA-Seq) instruments. Hi-C data were generated in the Tree of Life laboratory from remaining tissue of jaDiaLine7 using the Arima v2 kit and sequenced on a NovaSeq 6000 instrument.

### Genome assembly

Assembly was carried out with Hifiasm ( Cheng *et al.*, 2021); haplotypic duplication was identified and removed with purge_dups ( Guan *et al.*, 2020). One round of polishing was performed by aligning 10X Genomics read data to the assembly with longranger align, calling variants with freebayes ( Garrison & Marth, 2012). The assembly was then scaffolded with Hi-C data ( Rao *et al.*, 2014) using SALSA2 ( Ghurye *et al.*, 2019). The assembly was checked for contamination as described previously ( Howe *et al.*, 2021). Manual curation was performed using HiGlass ( Kerpedjiev *et al.*, 2018) and Pretext. The mitochondrial genome was assembled using MitoHiFi ( Uliano-Silva *et al.*, 2021), which performs annotation using MitoFinder ( Allio *et al.*, 2020). The genome was analysed and BUSCO scores generated within the BlobToolKit environment ( Challis *et al.*, 2020). Table 3 contains a list of all software tool versions used, where appropriate.

**Table 3.** Software tools used.

Software tool	Version	Source
Hifiasm	0.15.3-r339	Cheng *et al.*, 2021
purge_dups	1.2.3	Guan *et al.*, 2020
SALSA2	2.2	Ghurye *et al.*, 2019
longranger align	2.2.2	https://support.10xgenomics. com/genome-exome/ software/pipelines/latest/ advanced/other-pipelines
freebayes	1.3.1-17-gaa2ace8	Garrison & Marth, 2012
MitoHiFi	2.0	Uliano-Silva *et al.*, 2021
HiGlass	1.11.6	Kerpedjiev *et al.*, 2018
PretextView	0.2.x	https://github.com/wtsi-hpag/PretextView
BlobToolKit	3.0.5	Challis *et al.*, 2020

### Ethics/compliance issues

The materials that have contributed to this genome note have been supplied by a Darwin Tree of Life Partner. The submission of materials by a Darwin Tree of Life Partner is subject to the Darwin Tree of Life Project Sampling Code of Practice. By agreeing with and signing up to the Sampling Code of Practice, the Darwin Tree of Life Partner agrees they will meet the legal and ethical requirements and standards set out within this document in respect of all samples acquired for, and supplied to, the Darwin Tree of Life Project. Each transfer of samples is further undertaken according to a Research Collaboration Agreement or Material Transfer Agreement entered into by the Darwin Tree of Life Partner, Genome Research Limited (operating as the Wellcome Sanger Institute), and in some circumstances other Darwin Tree of Life collaborators.

## Data availability

European Nucleotide Archive: Diadumene lineata (orange-striped anemone). Accession number PRJEB46855; https://identifiers.org/ena.embl/PRJEB46855.

The genome sequence is released openly for reuse. The *D. lineata* genome sequencing initiative is part of the Darwin Tree of Life (DToL) project. All raw sequence data and the assembly have been deposited in INSDC databases. The genome will be annotated using the RNA-Seq data and presented through the Ensembl pipeline at the European Bioinformatics Institute. Raw data and assembly accession identifiers are reported in Table 1.

## Author information

Members of the Marine Biological Association Genome Acquisition Lab are listed here: https://doi.org/10.5281/zenodo.5913830.

Members of the Darwin Tree of Life Barcoding collective are listed here: https://doi.org/10.5281/zenodo.5744972.

Members of the Wellcome Sanger Institute Tree of Life programme are listed here: https://doi.org/10.5281/zenodo.6125027.

Members of Wellcome Sanger Institute Scientific Operations: DNA Pipelines collective are listed here: https://doi.org/10.5281/zenodo.5746904.

Members of the Tree of Life Core Informatics collective are listed here: https://doi.org/10.5281/zenodo.6125046.

Members of the Darwin Tree of Life Consortium are listed here: https://doi.org/10.5281/zenodo.5638618.
