# Supplementary material for: The genome sequence of the orange-striped anemone, Diadumene lineata (Verrill, 1869)
Source: Wellcome Open Res. 2022 Mar 15;7:93. [Version 1] doi: 10.12688/wellcomeopenres.17763.1 (PMC9975403; doi:10.12688/wellcomeopenres.17763.1)
Supplement: Peer review history

# Reviewer report: Reviewer response for version 1

**DOI:** 10.21956/wellcomeopenres.19660.r53407

**Published:** 2024-10-03

**Authors:** Kun Wang

**Affiliations:** 1 School of Ecology and Environment, Northwestern Polytechnical University, Xi’an, China

1. "it is now established on almost every temperate and tropical coast worldwide" might be overstated?

 2. "Under suitable conditions, it can quickly form large clonal aggregations." Should be more specified.

 3. "of the assembly sequence was assigned to 11 chromosomal-level scaffolds, representing 16 autosomes" is confusing.

 4. The range of DNA amount (0.01–0.5 ng) is unusually low for fragment size analysis. Should be explained.

Are sufficient details of methods and materials provided to allow replication by others?

Yes

Is the rationale for creating the dataset(s) clearly described?

Yes

Are the datasets clearly presented in a useable and accessible format?

Yes

Are the protocols appropriate and is the work technically sound?

Yes

Reviewer Expertise:

Evolutionary genetics

I confirm that I have read this submission and believe that I have an appropriate level of expertise to confirm that it is of an acceptable scientific standard.

# Reviewer report: Reviewer response for version 1

**DOI:** 10.21956/wellcomeopenres.19660.r53404

**Published:** 2023-01-03

**Authors:** Stacy Krueger-Hadfield, Will Ryan

**Affiliations:** 1 Department of Biology, University of Alabama at Birmingham, Birmingham, Alabama, USA; 2 Towson University, Towson, MD, USA

**Overall response: **Generating genomic resources for this species is a great step forward, which will facilitate a range of research for *D. lineata*, specifically, and sea anemones more broadly as genomic resources are not widespread at present.

 We provide some feedback below on the background, and to a certain extent the justification for sequencing this specific taxon, and on the methodology.

First, we provide some feedback that is geared toward improving the background information so that interested readers have a roadmap to the current literature on this species.

1. Both Flenniken (2017) and Glon et al. (2021) provided excellent background information on the invasion history and invasion-facilitating characteristics of *D. lineata*.
2. The link connected with the statement “Under suitable conditions, it can quickly form large clonal aggregations” leads to a non-functioning page. However, this statement could be supported with primary citations, such as:
  Shick (1976) which documented the occurrence of a high-density population, presumably of a single clone, in Blue Hills, Maine.
  Shick and Lamb (1977) which discussed the general tendency of *D. lineata *(under the former name *Haliplanella luciae*) to form clonal aggregations and provided allozyme evidence of clonal aggregations from a collection of sites.
  Ryan *et al. *(2021) which provided evidence with microsatellite markers of highly clonal population genetic structures from eight sites across the Atlantic Coast of the US.
3. Gamete production and mixed sex populations are now well established in the non-native range, though fertilization and larval settlement have not been confirmed in the field:
  Ryan and Miller (2019) demonstrated that gamete production is common across the range of *D. lineata* on the Atlantic Coast of the United States, including many populations with fertile males and females present.
  Newcomer *et al. *(2019) also found a population with fertile males and females coexisting on the Pacific Coast of the United States.
4. Gamete production in *D. lineata* has been documented in Japan (within the presumed native range) by Fukui (1991), Fukui (1995), and Ryan and Kubota (2016), but not Ryan and Miller (2019).
5. Asexual reproduction in *D. lineata* has been documented in Japan by several authors, including Uchida (1932, 1936) and Atoda (1973), but not Ryan and Miller (2019).
6. It might also be useful to acknowledge that literature on this species has been published under many species names over time. Hancock *et al.* (2017) gives a particularly nice overview of the taxonomic history and documented occurrences.

Second, we provide some feedback on the methodology to generate the genomic data.

1. In Figure 1, perhaps circle the specific specimen to which you are referring as there are multiple individual anemones in the photograph. Perhaps even cropping the image would be useful.
2. More information on the manual curation would be nice to determine exactly what was done. For example, was there any bacterial removal from the assembly?
3. Was RNA extracted from the whole organism?
4. Were both anemones – jaDialine6 and jaDialine7 – sterile? Also, when were they  collected (date?). The authors mention ‘unknown sex’ in the Genome sequence report section, but a little bit more information would be useful. Also, this is presumably from the sample used for HiC rather than RNA? Two anemones were used for all sequencing, so clarifying this is also useful.
5. Continuing from the point above, more information on what specimens were sequenced would be useful.

Are sufficient details of methods and materials provided to allow replication by others?

Partly

Is the rationale for creating the dataset(s) clearly described?

Partly

Are the datasets clearly presented in a useable and accessible format?

Yes

Are the protocols appropriate and is the work technically sound?

Yes

Reviewer Expertise:

Evolutionary ecology, population genetics, sea anemone biology

We confirm that we have read this submission and believe that we have an appropriate level of expertise to confirm that it is of an acceptable scientific standard.

## References

[1] : Pedal laceration of the sea anemone Haliplanella luciae. *P-SMBL* .1973;20:299-313

[2] : Embryonic and larval development of the sea anemone Haliplanella lineata from Japan. *Hydrobiologia* .1991;216-217(1) : 10.1007/BF00026454 137-142 10.1007/BF00026454

[3] : Seasonal changes in testicular structure of the sea anemoneHaliplanella lineata (Coelenterata: Actiniaria). *Invertebrate Reproduction & Development* .1995;27(3) : 10.1080/07924259.1995.9672449 197-204 10.1080/07924259.1995.9672449

[4] : Mediators of invasions in the sea: life history strategies and dispersal vectors facilitating global sea anemone introductions. *Biol Invasions* .2020;22(11) : 10.1007/s10530-020-02321-6 3195-3222 10.1007/s10530-020-02321-6 32837266 PMC7429141

[5] : A sea anemone of many names: a review of the taxonomy and distribution of the invasive actiniarian Diadumene lineata (Diadumenidae), with records of its reappearance on the Texas coast. *Zookeys* .2017; 10.3897/zookeys.706.19848 1-15 10.3897/zookeys.706.19848 29118617 PMC5674082

[6] : The Contribution of Clonality to Population Genetic Structure in the Sea Anemone, Diadumene lineata. *J Hered* .2021;112(1) : 10.1093/jhered/esaa050 122-139 10.1093/jhered/esaa050 33507264 PMC8355472

[7] : Morphotype distribution of the sea anemone Diadumene lineata in Tanabe Bay, Wakayama: a comparison with Uchida (1936) after 80 years. *P-SMBL* .2016;44:1-6

[8] : Ecological physiology and genetics of the colonizing actinian Haliplanella luciae. In (GO Mackie, ed): Coelenterate Ecology and Behavior. *Springer (Boston, MA, US)* .1976;137-146

[9] : ASEXUAL REPRODUCTION AND GENETIC POPULATION STRUCTURE IN THE COLONIZING SEA ANEMONEHALIPLANELLA LUCIAE. *The Biological Bulletin* .1977;153(3) : 10.2307/1540609 604-617 10.2307/1540609

[10] : Occurance in Japan of Diadumene luciae, a remarkable actinian of rapid dispersal (with plate IV, 1 chart and 4 text-figrures). *Journal of the Faculty of Science, Hokkaido Imperial University. Series VI, Zoology* .1932;2:69-82

[11] : Influence of the currents upon the distribution of races and frequency of asexual reproduction in actinian, Diadumene luciae[Japanese with English summary]. *Zoological Magazine, Tokyo* .1936;48:895-906

# Reviewer report: Reviewer response for version 1

**DOI:** 10.21956/wellcomeopenres.19660.r49216

**Published:** 2022-03-21

**Authors:** Craig Wilding

**Affiliations:** 1 School of Biological and Environmental Sciences, Liverpool John Moores University, Liverpool, UK

This Genome Note provides a useful review of the genome assembly and annotation efforts for *Diadumene lineata*. As there is currently a dearth of genome assemblies from the Cnidaria and particularly chromosome-level assemblies (perhaps only for the recent assemblies of *Nematostella vectensis* and *Scolanthus callimorphus *(Zimmerman *et al. *2022), ^1^ the addition of this assembly from *Diadumene lineata* is welcome.

 The genome size (313Mb) and chromosome number (N=16) is consistent with those of *N. vectensis* (244Mb, 15 chromosomes) and *S. callimorphus* (414Mb, 15 chromosomes) (Zimmerman *et al. *2022). ^1^

 It would be interesting to have further details of the manual curation that led to the welcome improvement in scaffold number and scaffold N50, and also useful to know what tissues were sampled for the RNASeq dataset. Presumably, this is the whole organism? However, the resources detailed within will be extremely useful for the study of cnidarian genomics and beyond.

Are sufficient details of methods and materials provided to allow replication by others?

Partly

Is the rationale for creating the dataset(s) clearly described?

Yes

Are the datasets clearly presented in a useable and accessible format?

Yes

Are the protocols appropriate and is the work technically sound?

Yes

Reviewer Expertise:

Genomics (invertebrates)

I confirm that I have read this submission and believe that I have an appropriate level of expertise to confirm that it is of an acceptable scientific standard.

## References

[1] : Sea anemone genomes reveal ancestral metazoan chromosomal macrosynteny. *bioRxiv* .2020; 10.1101/2020.10.30.359448 10.1101/2020.10.30.359448
